# Parent—youth interactions: Transitioning to toileting self-management in spina bifida patients

**DOI:** 10.1016/j.hctj.2023.100009

**Published:** 2023-07-14

**Authors:** Tae Kawahara, Akemi Yamazaki

**Affiliations:** Department of Pediatric and Family Nursing, Division of Health Sciences, Graduate School of Medicine, Osaka University, 1-7 Yamadaoka, Suita City, Osaka 565-0871, Japan

**Keywords:** Child, Incontinence, Parent, Spinal dysraphism, Transition

## Abstract

**Purpose:**

Spina bifida (SB) involves neurogenic bladder and bowel deficits. While parents manage the bladder and bowel disorders of their youth in the early years, the youth themselves must eventually take responsibility for their own management. However, the experience of shifting responsibility for complex toilet management from the parents to the youth has not been thoroughly investigated. Therefore, as exploratory research, the present study aimed to reveal the interactions between parents and youths with SB during the time of increasing responsibility for bladder and bowel management (i.e., the transition phase).

**Methods:**

Twelve parent–youth pairs (youths with SB aged 11–18 years) participated in dyadic interviews, and a parent–youth transition experience was categorized by the context of parent–youth interactions and analyzed using the grounded theory approach.

**Results:**

The results indicated that parents provide professional, complete bladder and bowel management until their youth reach physical and mental maturity. During the transitional phase, they work together to master youth self-management in interactions described as “share, try, and decide through parent–youth interactions”. Finally, the youth are able to master control of their own bladder and bowel management without requiring assistance from their parents.

**Conclusions:**

Although parent–youth interactions are not always present during the pre- and post-transition phases, the parent–youth relationship acts to facilitate the independence of the youth by taking advantage of the parent–youth subsystem during the transitional phase. Interventions during these challenging periods could help facilitate the transition to bladder and bowel self-management among the youth.

## Highlights

• Parents provide expert bladder and bowel management until the pre-transition phase.
• Unique parent–youth collaborations are central to the transitional toileting phase.
• Youths with SB master toilet self-reliance using adapted tools and others.

## 1 Introduction

Spina bifida (SB), a congenital disorder in which the neural tube does not close during fetal development, is associated with multiple complications, such as neurogenic bladder, hydrocephalus, and lower extremity sensorimotor deficits. In particular, the site of nerve damage is often higher than the nerves associated with the bladder and bowel, and 90% of children with SB eventually require clean intermittent catheterization (CIC). Furthermore, youths with SB often develop marked constipation or soft stool/diarrhea, the severity and frequency of which vary significantly between individuals.^1^ Youths with SB engage in a combination of different bowel management methods at home to facilitate a better social life.^2^ Notably, bladder and bowel management is commonly performed by parents of youths with SB in the early years,^3^ whereas developmental maturation eventually allows the youth to acquire independent bladder and bowel management.^4^

Youths with SB tend to depend on their parents, many of whom tend to be overprotective and over-involved in their youth’s lives.^5^ The parents who work together with youths with SB to manage bladder and bowel care have been found to structure their lives around everyday bladder and bowel management and unexpected incontinence,^6^ and tend to cooperate to hide bladder and bowel disorders from people around their youth.^7^ Parents are concerned that their youth with SB need help to complete self-management and may not be able to manage complex self-management, such as bowel control, on their own.^8^ Parents are thus more likely to direct their youth’s self-care, especially during puberty,^5^ and parental behavior and psychological control over youths with SB are known to affect their adherence to medical care.9., 10.

It has been noted that one-third of all youths with SB are able to live independently, while the remaining two-thirds are able to live independently with the help of others and the environment.^11^ In addition, the Self-Management and Independence Guideline^12^ implies the need for increased responsibility among youths with SB for bladder and bowel management during the transition to adulthood.

In practice, independent toilet management may not progress well among youths with SB, and the parent–youth interactions during the transition phase of bladder and bowel management, which affect the degree of independence with toilet management, remain poorly understood. Compared with Europe and the US, parent–child relationships in Japan are based on lifelong obedience to the parent to ensure family stability, and it is clear that as long as the cooperation and coexistence between parents and their youth are maintained for a long time, the parent–child attachment relationship remains stable, the parent–child relationship is closer, and the children maintain a close relationship with their parents both physically and psychologically.13., 14. Therefore, the transition experience of Japanese youths with SB and their parents could be expected to be characterized by deeply integrated parent–child interactions.

Given this background, the purpose of the present exploratory study was to reveal the interactions between Japanese parents and their youth with SB during the transitional phase of bladder and bowel management, which encompasses individuality.

## 2 Material and methods

### 2.1 Inclusion and exclusion criteria

This study involved youths with SB aged 11–18 years who were in a formal operational stage^15^ in which they could talk specifically to their parents about bladder and bowel management. The exclusion criteria were youths without a definite diagnosis of SB, youths unable to respond to interviews, parents and/or youths with SB who could not talk about their experiences in dyadic interviews, and youths with no history of either bladder or bowel management.

### 2.2 Dyadic interviews to capture the parent–youth subsystem

Dyadic interviews are conducted with two people who share a single event, and are often used in family research. This type of interview provides multiple dimensions of a shared event in the course of dialog between two people and helps provide a more accurate and in-depth context for the event, especially when there is an element of interaction between the two people that is difficult to observe or understand.^16^ Bladder and bowel management among youths with SB has been an openly shared act in families since birth, and while the parent or youth is the primary manager, the other also shares and evaluates the management process, thereby completing the youth’s bladder and bowel management through the parent–youth interaction. Thus, while defecation management in living spaces and the topic of defecation in open daily conversation are not taboo between parents and youths, there is a tendency to conceal bladder and bowel disorders in social life.^7^ For these reasons, it was considered that the interaction of parent–youth subsystems during the transition period could not be adequately observed in a single interview. Therefore, dyadic interviews of parents and youths were conducted with the aim of having them talk about their bladder and bowel management while supplementing and reminding each other of their personal stories. Although the interviews consisted of several questions by the interview guide, the participants were basically free to talk about their bladder and bowel management experiences. In the interviews, it was necessary to verbalize how the other perceived the stories told by one of the speakers and to encourage active conversation between the parent and youth, with the purpose of deepening the stories they had experienced. For this purpose, the interviewer repeated the speaker’s words, asked questions that evoked various scenes, and referred the conversation to the other speaker.

### 2.3 Data collection procedure

The inclusion/exclusion criteria were confirmed by carefully selecting participants based on their medical records in concert with a physician or physical therapist from two medical facilities and an attribute sheet completed during the interviews. To collect a well-balanced group of youths with SB with varying degrees of independence in terms of bladder and bowel management, referrers were asked for recommendations. In addition, people up to the age of 18 years who were well on their way to transitioning to bowel self-management and able to talk about those times in retrospect were recruited. Recruitment was conducted through two SB-related clinics and snowball sampling: 12 pairs of youths with SB and their parents were interviewed at home or elsewhere. Consent for this study was confirmed in writing and verbally at the study visit. Semi-structured interviews of dyads consisting of youths with SB and their parents were conducted to explore the characteristics of bladder and bowel management, a common event for parents and youths. The interviews and analyses were conducted in Japanese and translated into English. Questions to the parents and youths regarding who performs bladder and bowel management, how it is performed, and what they do in terms of bladder and bowel management explored the following research question (RQ): “What is your experience in the transitional phase of bladder and bowel management performed by parents and youths?”.

### 2.4 Analysis

The grounded theory approach (GTA) is a qualitative analysis method that aims to construct a theory from the process of abstracting and conceptualizing data using the properties and dimensions of a data-rooted limited sample.^17^ The data obtained in this study were recorded verbatim, and the context of the phenomenon and the part of the change were understood through the reading of data related to bladder and/or bowel management performed by youths with SB and their parents. The data were then interpreted at the parent–youth conversational level related to the RQ, properties and dimensions were extracted, and the interpreted data were labeled. Similar labels were merged to extract emergent categories, and as the number of interviews increased, properties and dimensions were reexamined and modified as appropriate. Based on the RQ, the phenomenon was paradigmatically categorized into situations, actions/interactions, and consequences, and categories of parent–youth interactions central to explaining this phenomenon were generated. In this study, labels and categories were confirmed through continuous comparisons between cases in each interview to depict the transitional phase of elimination management at a higher level of abstraction while maintaining individuality for the 12 youths with SB and their caregivers, who showed significant individual differences in elimination management. Interviews were completed with 12 pairs for whom the categories of parent–youth interactions during the transitional phase of elimination management had been confirmed. An analysis of the 12 interviews determined that data saturation had been achieved and sampling was complete.

### 2.5 Guarantee of plausibility and credibility

In this study, the first author was supervised by the second author (who is proficient in this research methodology) during each interview, the open coding, and the category elicitation stages. In addition, at the open coding stage in cases 1, 4, 6, 10, 11, and 12, data and analysis review meetings were held among university staff and graduate students familiar with the GTA to confirm the concepts and categories through an exchange of opinions on labels, category interpretations, and nomenclature. In the eleventh and twelfth cases, further data were collected to confirm the paradigm as a whole.

### 2.6 Ethical considerations

The Institutional Review Board of Osaka University approved the study protocol (Approval no. 17338). All parents provided written, informed consent. The study aims and methods were explained to the youths with SB, and whether they understood the requirements and agreed to participate was verified.

## 3 Results

### 3.1 Participants’ characteristics

The study participants were 12 pairs of parents and youths. The mean age ± standard deviation (SD) of the youths with SB was 15.75 ± 2.30 years, and five were boys. Among the participants, 11 youths performed CIC for bladder control, and the other one had continuous bladder incontinence. Eleven youths managed bowel control through a combination of laxatives, enemas, explants, ablutions, progressive enemas, and stoma management; the remaining youth did not need bowel management. The parents who participated in the interviews included nine pairs with mothers (one foster mother), one pair with a father, and two groups with both parents. The mean age ± SD of the parents was 47.54 ± 3.70 years. The mean interview time ± SD was 106 ± 19.44 min (Table 1). The interviews focused on the independence of bladder and bowel management. In the data-labeling phase, the parent–youth interactions common to bladder and bowel management were identified and integrated into subcategories and categories. Categories were extracted for one pre-transition phase, four transition phases, and one post-transition phase.

**Table 1:** Characteristics of the study participants.

ID	Sex	Age of youth with SB (years)	Attendees	Bladder management	Bowel management (frequency)	Main person (youth or parent) for bladder/bowel management	Time (min)
A	Girl	18	Father and mother	CIC	Stoma (1–2 times/day)	Youth/youth	90
B	Boy	15	Mother	Diaper, pad, CIC	Enema (1 time/2–3 days)	Youth/both	112
C	Boy	17	Father and mother	CIC	Transanal irrigation (irregular)	Youth/both	88
D	Girl	11	Mother	CIC	Laxatives and transanal irrigation (1 time/2 days)	Both/parent	128
E	Girl	17	Mother	CIC	Enema (1 time/1–2 days)	Youth/parent	97
F	Girl	17	Mother	CIC	Laxatives (every day), enema (3 times/week), stool extraction (5 times/week)	Youth/both	102
G	Girl	16	Mother	Diaper, pad, CIC	Enema and transanal irrigation (every day)	Youth/both	118
H	Boy	12	Mother	CIC	Stool extraction (every day)	Both/parent	140
I	Boy	17	Father	CIC	Laxatives (every day), enema (1 time/3 days), transanal irrigation (irregular)	Youth/youth	92
J	Girl	14	Mother	CIC	Stool extraction (every day)	Both/youth	80
K	Boy	18	Mother	Diaper, pad	Stool extraction and strain at bathroom (every day)	Both/parent	121
L	Girl	17	Mother	CIC	No bowel management	Youth/youth (no bowel management)	85

CIC = clean intermittent catheterization.

### 3.2 Youths with SB and their experiences of the transitional phase of bladder and bowel management performed with their parents

**Fig. 1:**
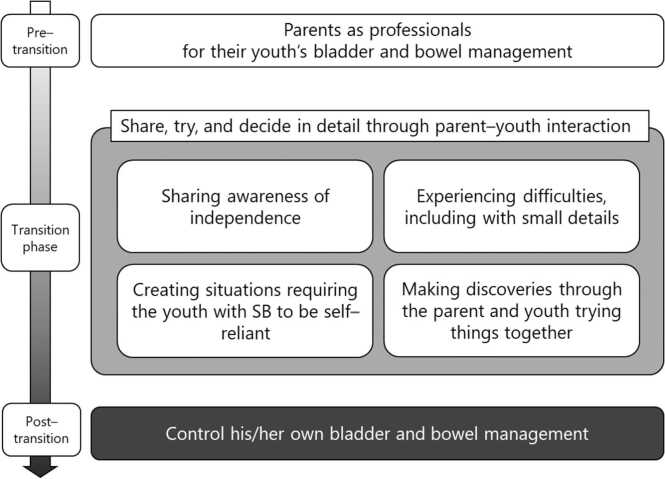
Paradigm of the transitional structure of bladder and bowel management as perceived by parents and youths. In the pre-transitional phase, parents mainly manage their youth’s toileting, in the transitional phase, parents and youths work together cooperatively, and in the post-transitional phase, youths with SB mainly manage their toileting on their own. “Share, try, and decide in detail through parent–youth interactions” is representative of the four parent–youth interactions in the transition phase.

Fig. 1 shows a paradigm of the transitional structure of bladder and bowel management from the perspective of parents and youths. Youths with SB require bladder and bowel management from early childhood, and to ensure appropriate CIC and bowel management, “***Parents act as professionals for their youth’s bladder and bowel management***”. During the transition phase, there were four interactions, described collectively as “***share, try, and decide in detail through parent–youth interactions***”. In the transition phase, parents and youth ***“shar***ed ***awareness of independence***” to set specific goals for their youth’s self-management and “***experienc***ed ***difficulties**, **including with small details***” in the process of clarifying which parts they could do on their own and which parts they needed help from the environment and/or others. In addition, they tried “***creating situations requiring the youth with SB to be self-reliant***” and consciously reduced parental help. They repeated “***making discoveries through the parent and youth trying things together***” to adapt tools and methods to cope with what they could not do by themselves, and changed the method of toileting. Through these repeated experiences, youths with SB were able to “***control his/her own bladder and bowel management***”, i.e., independent bladder and bowel management for youths with SB, and built a structure to use the toilet by themselves independently.

#### 3.2.1 Parents as professionals for their youth’s bladder and bowel management (pre-transition)

Although youths with SB had required bladder and/or bowel management since childhood, they tended to have inadequate self-management owing to “ambiguous sensations in the buttocks” and the “limitations caused by physical disabilities”. For this reason, it was essential for parents to act as professionals and take over bladder and bowel management for their youths. Parents coordinated daily bladder and bowel management with medical care providers at their child’s school and assisted their youths with SB in dealing with fecal incontinence due to uncontrollable bowel movements.

*I have been performing his bowel management for many years, so I know the state of his bowels. I put in a full bottle (of enema solution), wait with the stopper for 5 min, and then his stool comes out and he can go for 2 days without any bowel incontinence. (Mother B)*

CIC was performed every 2–4 h as bladder management, and for youths with SB, bladder management meant “time-determined CIC”.

*I have to be mindful of the time (for CIC) even when I am playing with friends, and I also have to be concerned about the smell (of bladder incontinence) because I don’t feel incontinent. I can’t do what I want at the time I want, and the set time is quite difficult for me. (Youth J)*

With parental or substitute management by other adults, youths with SB and their parents tended not to experience difficulties because of constant assistance and proactive handling.

*When asked, “Do you have any problems?”, she said, “Nothing in the end.” (Mother K)*

*People around me help a lot, so I don’t really have any problems. (Youth K)*

*She has been living her life as a thinker from birth, so she might not really find it inconvenient. (Mother K)*

*I have never thought that (my body) is inconvenient. (Youth K)*

#### 3.2.2 Share, try, and decide through parent–youth interactions (during the transitional phase)

##### 3.2.2.1 Sharing awareness of independence

Parents and youths used to take for granted that parents would take care of their youth’s bladder and bowel care, but both parents and youth came to recognize and share the need for the youth’s independence at the time of events occurring when the youth left the parent, such as school trips, entering a school or dormitory, or becoming a member of society. Whereas caregivers consider “independent” bladder and bowel management to be planned bladder and bowel care that does not affect daily life, youths with SB tend not to be able to have concrete conceptions of what “independence” is. Therefore, parents and youth believed that it was necessary to reconcile their view of the goal of independence step-by-step.

*The school trip was coming up at just the right time, and… (Mother A)*

*We agreed, “Now is a good time.” (Youth A)*

*I guess the reason for doing it then was the school trip. (Mother A)*

*Yeah, that was big. (Youth A)*

##### 3.2.2.2 Experiencing difficulties, including with small details

Youths with SB may not be able to perform self-management as well as their parents. In these situations, they experience both successful and unsuccessful experiences repeatedly. They and their parents shared the difficulty and developed a pattern for solving problems in detail one-by-one and accumulated experiences together. The problems for youths with SB were hanging water bottles for irrigation in high places, maintaining posture, holding tools while using them, and feeling sensations around the pubic zone.

*I thought I would let her practice a bit with managing her own bowel movements, so I bought one of those Ichijiku enema kits, the ones that are sold in shops. (Mother E)*

*I couldn’t see my butt. (Youth E)*

*I got her to try it because I thought if she could reach her bottom and then just push firmly, she could probably do it. The bit that goes in is only about this long as well, and I didn’t think there would be a problem. But she couldn’t even insert it into her anus properly. (Mother E)*

##### 3.2.2.3 Creating situations requiring the youths with SB to be self-reliant

As parents are tempted to intervene in the youth with SB’s self-management, and as youths with SB wish to rely on their parents, parents and youths with SB consciously created a situation in which the youth with SB had to perform self-management alone without parental help. In this situation, even if the youth’s techniques and management were not perfect, the caregivers tolerated them as long as they did not cause physical problems.

*If she is incontinent at home, I tell her to do the rest on her own and I leave her alone. She wipes her own buttocks and changes her own diapers. If she asks me to help her with something she can’t do, I help her. Otherwise, I leave her alone. (Mother E)*

*But I don’t tell her. (Youth E)*

*I have to harden my heart and pretend I don’t see it. (Mother E)*

##### 3.2.2.4 Making discoveries through the parent and youth trying things together

Youths with SB matured by using methods and techniques in the process of becoming independent in their management. They sought out applications and innovations one-by-one to identify ways to make self-management easier and more successful, and then shared the results with their parents. On the other hand, youths with SB and their parents had the flexibility to try new ways of self-management that they found difficult for the youth with SB to complete themselves. The most common technique for youths with SB was CIC, and many youths with SB felt that they were not comfortable with bowel management, which involves many difficulties. They had given up on “probing the anus”, “hanging a bottle on the wall and pouring hot water down it”, and “lifting the buttocks”, choosing instead to “have someone insert the liquid into the anus”, “use a bottle on the floor”, and “create a Malone antegrade continence enema”.

*We’ve tried various things. We administer [the antegrade continence enema fluid] with a syringe, but we were wondering whether to try using a top-loading bag instead, but I don’t know because that takes more time. So for now, we have decided that the syringe is probably better after all. But [in the dorm] it’s not like the nurse can be there all the time either, so if she’s holding it in, she’s only got one hand free. It’s the nurse who administers (the antegrade continence enema fluid). (Mother G)*

*When I’m at the dorm, the syringe is already completely filled [with the fluid]. (Youth G)*

*She usually holds the stoma [anal plug] in her bottom. She moved into the dorm after practicing using the syringe to administer it herself and […] making sure she was able to do it herself if someone else would hold it in for her. (Mother G)*

#### 3.2.3 Control his/her own bladder and bowel management (post-transition)

Youths with SB recognized their own various limitations down to the smallest details and understood the range of their ability to perform defecation on their own. In the parts exceeding their own capabilities, they were able to utilize “others as resources” and complete their defecation even in the absence of their parents. In addition, they began to plan ahead for bladder and bowel management so that their defecation problems would not interfere with their social life.

*I think (independence) is knowing the limits of what I can do by myself. “Independence” might be understanding the extent to which I can do it by myself, and then being able to ask others for help when I am not able to do it by myself. (Youth C)*

*Others? (Interviewer)*

*The people around me. (Asking for help) I don’t have that much hesitation (Youth C)*

## 4 Discussion

To live at home with a youth who requires medical care, caregivers need to master appropriate care for their youth’s condition and integrate it into their lifestyle.^18^ As described in the themes, “***Parents act as professionals for their youth’s bladder and bowel management***” because they have alternative management methods for their youth’s bladder and bowel disorder and master specialized techniques for their conditions, similar to medical professionals, by repeatedly managing their youth’s bladder and bowel needs. Although the Spina Bifida Association^19^ advocates that parents help their children with only what they need to promote independence, it has been shown that parents of youths with SB, especially those at younger ages, tend to be overprotective.^5^ The youth in the present study did not experience any specific difficulties caused by their disabilities, and all tasks were solved by the parents or adults ahead of time. These results are highly characteristic of dependent parent–child relationships in Japan,13., 14. and indicate that a lack of difficulties may not lead to a youth’s desire for independence because the experience of difficulties was extracted in the transition phase.

Previous research on the development of self-management in youths with chronic conditions has shown that the development of self-management is a gradual process of adherence to medical care, which is accompanied by the maturation of the youth’s own understanding of medical knowledge and other factors.^20^ O’Hara and Holmbeck^9^ found that adherence decreased in youths with SB when they attempted to transition at a time when they were not quite mature enough to do so. It has also been shown that psychosocial maturity is important for youths with SB to acquire self-management,^4^ and the youth’s increasing desire for independence is an important factor among parents and youths to promote independence. In the present study, parents and youths “***shared awareness of independence***” as part of the maturation process, and set their life events at a good time to achieve independence. Graven et al.^21^ showed that problem-solving skills are effective in enhancing self-management skills. In the present study, problem-solving skills were fostered through two interactions: “***experiencing difficulties, including with small details***” for youths with SB one-by-one through repeated experiences of self-management, and “***making discoveries through the parent and youth trying things together***” to deal with them. In the process of becoming accustomed to their own toileting techniques, youths with SB acquired their own unique applications and devices. Youths with SB have various limitations and learn to understand what they cannot do on their own. In these situations, they study how to balance habituation and achieve adequate bladder and bowel control. However, a previous study reported that finding the appropriate bowel program for the family can cause stress.^22^ Parents of youths with SB have been found to be prone to interfere in the self-management of youths with SB, especially during puberty and adolescence.^5^ In the present study, parents also resisted the impulse to interfere in the youth’s self-management, and parents and youths made a conscious effort to avoid intervening with each other by “***creating situations requiring the youth with SB to be self-reliant***”. Many parents in previous studies felt that bowel self-management programs are lacking^6^; thus, an environment in which parents and youths can separate and gain experience in self-management is needed.

Sliwinski et al.^23^ made it clear that when establishing self-management, it is important to recognize that the decision-maker for medical care, etc., is oneself. In the present study, youths with SB who had advanced to the independent stage also planned bladder and bowel management to not interfere with their daily life at school and with their friends, and understood when they should ask others for help, while their parents showed a trusting and non-interfering attitude toward their self-management. Although the youth with SB did not have a concrete image of independence at the beginning of the transition, they eventually realized that independence for them meant to “***control his/her own bladder and bowel management***”. Previous research has reported that positive efforts by health-care providers and families can promote independence in bowel behaviors,^24^ and that proactive transitional support is necessary for youths with SB and their parents. Bowel management is especially likely to be difficult and fraught with failure, so it is better to have an environment where one can easily consult a health-care professional. The strategic interventions for the four transitional parent–youth interactions defined in this study show potential for improving parent–youth synergy and promoting bladder and bowel management independence among youths with SB.

## 5 Implications for practice

In situations involving “***sharing awareness of independence***”, the image of independence in self-management perceived by the youth with SB and their parents may not always coincide. Therefore, health-care providers should take the opportunity to discuss specific self-management goals, such as “When do you want to be able to do and by when?” with the parent and youth. In the process of experiencing difficulties, youths with SB and their parents may experience feelings of inferiority and anxiety that may be intensified by the experience of failure. Health-care providers should emphasize that “***experiencing difficulties, including with small details***” means gaining the ability to imagine the concept of self-management and objective judgment, and then help the youths avoid converging on a complex. In addition, taking advantage of the interaction of “***making discoveries through the parent and youth trying things together***”, health-care providers should suggest various creative ways and new management methods so that the youth can accumulate successful experiences instead of sticking to failed ones. In “***creating situations requiring the youth with SB to be self-reliant***”, it is possible to change the conventional toilet environment that has been arranged to make it easier for the parents to perform. This change could promote an environment that actually facilitates the youth to perform toileting alone, and “***creating situations requiring the youth with SB to be self-reliant***” would facilitate such an environment.

## 6 Limitations and future research

The participants in the present study were selected based on their estimated level of independence in bladder and bowel management according to their medical records and visits, so patients who had not had a medical visit or appointment in at least the past 6 months to 1 year or participants who had not had consistent medical visits were excluded. In addition, because the interviews were designed to extract interactions through parent–youth conversations, patients were introduced according to good parent–youth relationships as determined by the medical staff. Therefore, the participants recruited in this study had relatively good parent–youth relationships and sufficient medical compliance to be able to visit the hospital regularly. Therefore, the results may not be applicable to those who do not have a good parent–youth relationship (e.g., those with typical conflicts between parents and youths during puberty). In future research, recruiting from local schools rather than hospitals and interviewing parents and youths whose relationships are not dependent or who do not regularly visit a health-care provider would likely reveal a more holistic transition structure.

## 7 Conclusions

During the early years, there was little parent–youth interaction and the youths with SB were dependent on their parents, who performed the youth’s bladder and bowel management perfectly. However, the following four parent–youth relationship themes emerged that summarized this transition phase of bladder and bowel management: “***sharing awareness of independence***”, “***experiencing difficulties, including with small details***”, “***creating situations requiring the youth with SB to be self-reliant***”, and “***making discoveries through the parent and youth trying things together***”. In conclusion, nursing interventions for parent–youth interactions specific to the transitional phase should help facilitate the youth’s self-management more effectively.

## Funding

This work was supported by grants from the Yamaji Fumiko Nursing Research Fund and the Japan Spina Bifida and Hydrocephalus Research Foundation (Grant no. 103). The funders of the study had no role in the design of the study; the collection, analysis, or interpretation of data; the writing of the report; or in the decision to submit the article for publication.

## CRediT authorship contribution statement

**Tae Kawahara:** Conceptualization, Methodology, Investigation, Software, Data curation, Writing – original draft, Funding acquisition. **Akemi Yamazaki:** Writing – review & editing.

## Declaration of Competing Interest

The authors declare the following financial interests/personal relationships which may be considered as potential competing interests: Tae Kawahara reports financial support was provided by The Yamaji Fumiko Nursing Research Fund. Tae Kawahara reports financial support was provided by The Japan Spina Bifida and Hydrocephalus Research Foundation.

## Data Availability

The data that has been used is confidential.

## References

[1] Freeman K.A., Castillo H., Castillo J. (2017). Variation in bowel and bladder continence across US spina bifida programs: a descriptive study. J Pediatr Rehabil Med.

[2] Kelly M.S., Wiener J.S., Liu T. (2020). Neurogenic bowel treatments and continence outcomes in children and adults with myelomeningocele. J Pediatr Rehabil Med.

[3] Ambartsumyan L., Rodriguez L. (2018). Bowel management in children with spina bifida. J Pediatr Rehabil Med.

[4] Davis B.E., Shurtleff D.B., Walker W.O. (2006). Acquisition of autonomy skills in adolescents with myelomeningocele. Dev Med Child Neurol.

[5] Holmbeck G.N., Coakley R.M., Hommeyer J.S., Shapera W.E., Westhoven V.C. (2002). Observed and perceived dyadic and systemic functioning in families of preadolescents with spina bifida. J Pediatr Psychol.

[6] Choi E.K., Ji Y., Bae E., Jang M. (2019). Parents’ needs concerning their children with spina bifida in South Korea: a mixed method study. J Pediatr Nurs.

[7] Moore C., Kogan B.A., Parekh A. (2004). Impact of urinary incontinence on self-concept in children with spina bifida. J Urol.

[8] Yun H.J., Choi E.K., Han S.W. (2021). Parents’ perception of self-management behaviors for their children with spina bifida in South Korea: a qualitative study. Rehabil Nurs.

[9] O’Hara L.K., Holmbeck G.N. (2013). Executive functions and parenting behaviors in association with medical adherence and autonomy among youth with spina bifida. J Pediatr Psychol.

[10] Psihogios A.M., Holmbeck G.N. (2013). Discrepancies in mother and child perceptions of spina bifida medical responsibilities during the transition to adolescence: associations with family conflict and medical adherence. J Pediatr Psychol.

[11] Oakeshott P., Hunt G.M. (2003). Long-term outcome in open spina bifida. Br J Gen Pract.

[12] Logan L.R., Sawin K.J., Bellin M.H., Brei T., Woodward J. (2020). Self-management and independence guidelines for the care of people with spina bifida. J Pediatr Rehabil Med.

[13] Rothbaum F., Pott M., Azuma H., Miyake K., Weisz J. (2000). The development of close relationships in Japan and the United States: paths of symbiotic harmony and generative tension. Child Dev.

[14] López-de-la-Nieta O., Koeneke H.M., Martinez-Rubio J.L., Shinohara K., Esposito G., Iandolo G. (2021). Exploration of the Spanish version of the attachment style questionnaire: a comparative study between Spanish, Italian, and Japanese culture. Eur J Investig Health Psychol Educ.

[15] Piaget J. (1964). Cognitive development in children: development and learning. J Res Sci Teach.

[16] Sohier R. (1995). The dyadic interview as a tool for nursing research. Appl Nurs Res.

[17] Corbin J.M., Strauss A. (2008). Basics of Qualitative Research: Techniques and Procedures for Developing Grounded Theory.

[18] Nishigaki K., Kanamori Y., Ikeda M., Sugiyama M., Minowa H., Kamibeppu K. (2016). Changes in mothers’ psychosocial perceptions of technology-dependent children and adolescents at home in Japan: acknowledgement of children’s autonomy. Asian Nurs Res.

[19] Spina Bifida Association. Guidelines for the care of people with spina bifida; 2018. 〈http://www.spinabifidaassociation.org/guidelines/Spina-Bifida-2018.pdf〉; [Accessed 16 February 2023].

[20] Nicholas D.B., Kaufman M., Pinsk M., Samuel S., Hamiwka L., Molzahn A.E. (2018). Examining the transition from child to adult care in chronic kidney disease: an open exploratory approach. Nephrol Nurs J.

[21] Graven L.J., Gordon G., Keltner J.G., Abbott L., Bahorski J. (2018). Efficacy of a social support and problem-solving intervention on heart failure self-care: a pilot study. Patient Educ Couns.

[22] Sawin K.J., Thompson N.M. (2009). The experience of finding an effective bowel management program for children with spina bifida: the parent’s perspective. J Pediatr Nurs.

[23] Sliwinski S.K., Gooding H., de Ferranti S. (2017). Transitioning from pediatric to adult health care with familial hypercholesterolemia: listening to young adult and parent voices. J Clin Lipidol.

[24] Seeley A., Lindeke L. (2017). Developing a transition care coordination program for youth with spina bifida. J Pediatr Health Care.

